# Low lysosomal acid lipase activity is associated with histological progression of metabolic dysfunction-associated steatotic liver disease

**DOI:** 10.1007/s12072-026-11084-6

**Published:** 2026-04-22

**Authors:** Rosa Lombardi, Felice Cinque, Annalisa Cespiati, Cristina Bertelli, Giuseppina Pisano, Giovanna Oberti, Erika Fatta, Jaqueline Currà, Emma Calzavara, Francesca Alletto, Carola Garavaglia, Giulia Cincotto, Anna Ludovica Fracanzani, Monica Gomaraschi

**Affiliations:** 1 Dipartimento di Fisiopatologia Medico-Chirurgica e dei Trapianti, Università degli Studi di Milano, Via F. Sforza, Milano, Italy; 2 SC Medicina Indirizzo Metabolico, Fondazione IRCCS Ca‘ Granda Ospedale Maggiore Policlinico di Milano, Milano, Italy; 3 Centro E. Grossi Paoletti, Dipartimento di Scienze Farmacologiche e Biomolecolari, Università degli Studi di Milano, Via Balzaretti 9, Milano, Italy

**Keywords:** Liver biopsy, Hepatic inflammation, MASH, Hepatic fibrosis, Fibroscan, Liver stiffness measurement, Liver injury, Disease progression, Lipid metabolism, Biomarker

## Abstract

**Background and purpose of the study:**

Liver inflammation and fibrosis are the key determinants of long-term adverse outcomes in metabolic dysfunction-associated steatotic liver disease (MASLD). Therefore, identifying progressive forms of MASLD is crucial. Lysosomal acid lipase (LAL), a central enzyme in intracellular lipid hydrolysis, has been reported to be reduced in MASLD and linked to liver injury.

**Aim:**

To evaluate whether baseline LAL activity predicts long-term progression of MASLD.

**Methods:**

We prospectively followed up 144 adults with biopsy-proven MASLD for a minimum of 5 years (median 8.1). All patients underwent a FibroScan® at baseline and follow-up and 94 of them also had paired liver biopsies. LAL activity (expressed as LAL/platelet-LAL/ptls) was measured on dried blood spots. Liver disease progression was defined by meeting of the histological endpoint at follow-up (i.e., development of MASH and/or NAS worsening ≥ 1 point and/or fibrosis progression ≥ 1 stage) or by liver stiffness measurement (LSM) worsening at Fibroscan.

**Results:**

Among the 94 biopsied patients, 26% met the composite histological endpoint and had significantly lower baseline LAL/ptls ratio, higher HOMA-IR, CAP and LSM. In particular, lower LAL/ptls was strongly related to the worsening of inflammation. In multivariate analysis, lower ln-transformed LAL/ptls ratio independently predicted histological progression (OR 0.51, 95% CI 0.04–0.60, *p* = 0.018). No association emerged between baseline LAL/ptls ratio and non-invasive progression by FibroScan.

**Conclusions:**

Lower LAL/ptls ratio identifies MASLD patients at increased risk of histological progression and complements non-invasive tools by reflecting biological pathways linked to inflammation.

**Graphical Abstract:**

Progression of MASLD and levels of Lysosomal acid lipase activity

**Figure:**
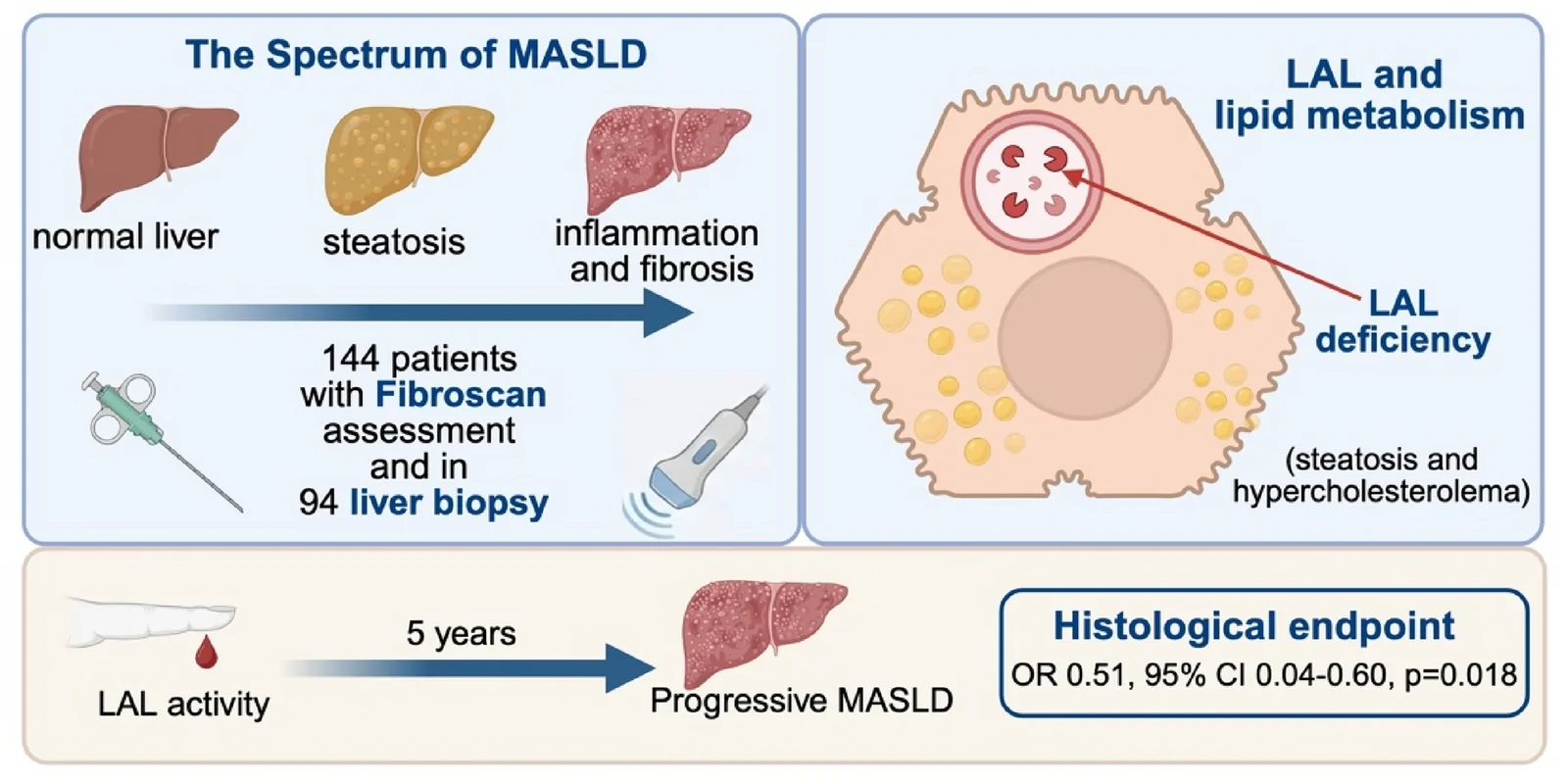

**Supplementary Information:**

The online version contains supplementary material available at 10.1007/s12072-026-11084-6.

## Introduction

Metabolic dysfunction-associated steatotic liver disease (MASLD) is defined as excess hepatic lipid accumulation in the presence of at least one cardiometabolic risk factor, and its prevalence is estimated to be around the 30% in the general adult population [1]. While simple steatosis is a benign condition, the progression toward metabolic–dysfunction-associated steatohepatitis (MASH), characterized by hepatic inflammation, and fibrosis lead to a dramatic increase in morbidity and mortality, representing a major public health concern [2]. Therefore, the identification of MASLD patients at higher risk of progressive disease is of utmost importance.

Liver biopsy remains the gold standard to define the severity of liver disease, but non-invasive tests are increasingly recognized as possible tools for the detection of the severity of liver disease, despite none of them is validated for the detection of hepatic inflammation [1, 3]. Among this, vibration-controlled transient elastography (VCTE) (i.e., Fibroscan) is an established technique easily applicable in standard clinical settings, providing an estimation of steatosis and fibrosis through two parameters called controlled attenuation parameter (CAP) and liver stiffness measurement (LSM), respectively [1, 4].

Lysosomal acid lipase (LAL) is the enzyme responsible for the hydrolysis of cholesteryl esters and triglycerides in the lysosomal compartment [5]. Generated unesterified cholesterol and free fatty acids move to the cytosol, where they can regulate their own synthesis and metabolism. Lipids can reach the lysosomes by receptor-mediated endocytosis of circulating lipoproteins or by autophagy-guided transfer of lipid droplets (LD) from the cytosol. Indeed, accumulating evidence in different cell types indicates the relevance of lysosomal hydrolysis over the cytosolic one by neutral lipases for LD clearance [6, 7]. Furthermore, the key role for LAL in cellular and systemic lipid metabolism is demonstrated by the phenotype of patients with genetic LAL deficiency (LAL-D), characterized by liver steatosis and hypercholesterolemia [8]. In searching for probands of genetic LAL-D, data in literature have previously shown that patients with MASLD can develop an acquired LAL-D [9, 10]. This was confirmed by a previous study from our group in a cohort of biopsy-proven MASLD patients, who presented significantly lower levels of LAL activity compared to dyslipidemic patients. Moreover, while LAL activity values were below normal values on average, a great interindividual variation was observed, with 36% of patients having normal LAL values [10]. Based on these premises, aim of the present study was to prospectively assess whether LAL activity levels could predict MASLD progression over time.

## Materials and methods

### Patients cohort

A total of 144 patients with biopsy-proven MASLD were followed longitudinally for a minimum of 5 years (median 8,1 years, IQR 7,6–8,8) at the outpatient clinic of Metabolic and Liver Disease. As recommended by International Guidelines, all patients were offered lifestyle changes, including dietary advice and recommendations for physical activity [11]. At enrollment, patients underwent determination of LAL activity. Both at baseline and follow-up patients underwent assessment of hepatic disease by Fibroscan. In addition, 94 out of 144 enrolled patients underwent paired liver biopsy (i.e., baseline and follow-up) for clinical reasons (i.e., increased LSM values, persistently altered transaminases, increase in serum ferritin, long-lasting history of steatosis, and multiple metabolic comorbidities).

Exclusion criteria were assumption of steatosis-inducing drugs (glucocorticoids, amiodarone, tamoxifen, methotrexate, valproic acid and nimesulide etc.), coexistence of viral hepatitis, HIV infection, Wilson’s disease, alpha1-antitrypsin deficiency, autoimmune hepatitis, genetic hemochromatosis and excessive alcohol consumption (> 20 g in women, > 30 g per day in men).

The study protocol was approved by the Institutional Review Board of Fondazione IRCCS Ca’ Granda Ospedale Maggiore Policlinico of Milan. For all patients, informed consent to use clinical, histological and laboratory data was obtained across decades, according to the ethical guidelines of the 1975 Declaration of Helsinki.

### Assessment of anthropometric features and metabolic comorbidities at enrolment

All clinical data and blood samples were collected both at enrollment and at follow-up.

Patients were classified as normal weight, overweight and obese according to body mass index (BMI) values of < 25, 25–29.9 and > 30 kg/m^2^, respectively. Hypertension was defined by in-office blood pressure ≥ 140/90 mmHg or use of any antihypertensive drugs, whereas dyslipidemia was defined by LDL-cholesterol > 100 mg/dL (or lower according to the SCORE2 cardiovascular risk), triglycerides > 150 mg/dL and/or HDL-cholesterol < 40 mg/dL for men and 50 mg/dL for women, or use of lipid-lowering drugs, according to international guidelines [12, 13]. In addition, type 2 diabetes (T2DM) was defined by the detection of a serum fasting glucose ≥ 126 mg/dL in at least two determinations and/or ≥ 200 mg/dL at a random determination and/or after an oral glucose load and/or a glycated hemoglobin ≥ 48 mmol/mol. Homeostatic model assessment insulin resistance index (HOMA-IR) > 2.5 was diagnostic for insulin resistance [14]. Current therapy was enquired, including the use of any hypoglycemic and lipid-lowering drugs. A complete biochemical panel including glucose and lipid parameters was assessed in all patients.

### Measurement of LAL activity

LAL activity was measured on dried blood spots (DBS) by fluorescence, using 4-methylumbelliferone palmitate (4-MUP, Cayman Chemical), cardiolipin (Avanti Polar Lipids) and the selective LAL inhibitor Lalistat 2 (Merck), according to the method of Hamilton et al. [10, 15]. Briefly, a 3.2 mm blood spot was eluted in H_2_O and incubated with or without 30 mol/l Lalistat-2. Then, 0.15 mol/l acetate buffer pH 4.0 with 1% Triton X-100, cardiolipin and 4-MUP was added and the mix was incubated at 37 °C. Generated 4-methylumbelliferone (4-MU) was detected by the Synergy H1 Multi-Mode microplate reader and GEN5 software (BioTek) with excitation at 320 nm and emission at 460 nm. A standard curve of 0–2.5 nmol 4-MU (Sigma-Aldrich) was built. LAL activity was calculated by subtracting the activity in the inhibited reaction (with Lalistat 2) from uninhibited reaction (with H_2_O) and expressed as nmol of generated 4-MU/spot/h. LAL activity on DBS is strongly correlated with platelet count [10]. Since platelets can decrease during liver disease progression, LAL activity was also expressed as LAL/platelets ratio (LAL/ptls). Laboratory personnel performing LAL measurements was blinded to patients’ clinical data and outcomes.

### Liver assessment

FibroScan® was performed by one expert physician who was blinded to participants’ clinical details, using the M probe or the XL probe in case of unsuccessful measurement, and the final value was obtained using standardized procedures. According to literature consensus, controlled attenuation parameter (CAP) value ≥ 280 defined the presence of severe liver steatosis, whereas a liver stiffness measurement (LSM) value ≥ 8 defined the presence of advanced liver fibrosis (≥ F3) [11]. Advanced chronic liver disease (ACLD) was defined by LSM ≥ 15 kPa according to Baveno VII consensus or in the presence of clinical/ultrasound evidence of cirrhosis [16].

As mentioned above, 94 out of 144 patients underwent liver biopsy at baseline and follow-up.

A single pathologist reviewed the biopsy samples and the Kleiner classification was used to grade steatosis, lobular inflammation, and hepatocellular ballooning, and to stage fibrosis from 0 to 4 [17]. The NAFLD activity score (NAS) was graded from 0 to 8, on a scale, including additional scores for steatosis, lobular inflammation, and hepatocellular ballooning [17]. The presence of MASH was based on the presence of NAS ≥ 5 including at least 1 point for ballooning. The liver pathologist was blinded to patients’ clinical data and outcomes.

Serum levels of transaminase > 39 U/L and ≥ 41 U/L for AST and ALT, respectively, and gamma-glutamyltransferase (GGT) ≥ 61 UI for men and > 36 UI/l for women, respectively, were considered elevated, according to local laboratory cutoffs. Transaminases, along with age and platelet count, were included in the fibrosis-4 (FIB-4) formula.

Information on alcohol consumption and smoking habits was also collected.

### Statistical analysis

Continuous variables were expressed as mean ± standard deviation or median (interquartile range, IQR). When needed, correlation of parameters was investigated by calculating the Spearman rho coefficient of correlation. Categorical variables were analyzed as absolute and relative frequencies (n, %). The chi-squared test (for categorical variables), the unpaired Student’s *t* test and the Mann–Whitney test (for normally and not normally distributed continuous variables, respectively) were used to compare differences between the groups. LAL activity and LAL/ptls ratio were logarithmically transformed (Ln) for the analysis. The LAL/ptls ratio was also analyzed according to tertiles when appropriate.

To evaluate the temporal changes in liver parameters from baseline to follow-up the paired Student’s *t* test and the independent sample proportions were used for continuous and categorical variables, respectively. Multivariable logistic regression analysis was also performed to identify significant predictive factors of liver disease progression. Progression was defined as worsening of CAP and LSM, worsening of inflammation expressed by NAS, development of MASH, worsening of fibrosis and as the occurrence of a composite histological endpoint (defined as worsening of NAS and/or development of MASH and/or worsening of fibrosis), after adjusting for common metabolic risk factors and other covariates that were statistically significant at univariable regression analyses.

Receiver operating characteristic (ROC) curve analysis was performed to evaluate the discriminative ability of a model including variables significantly associated with worsening of the histological endpoint or with intrinsic physiological impact on it. The incremental predictive value of LAL/ptls was assessed by comparing the area under the curve (AUC) of the model with or without the incorporation of LAL/ptls.

Missing data were assessed for extent and pattern. When the proportion of missing values was < 5% analyses were performed using a complete-case approach [18].

A two-tailed *p* value ≤ 0.05 was considered statistically significant. All data were analyzed using the statistical package IBM SPSS software 29.0 (Chicago, IL).

## Results

### Baseline features of the whole cohort

As reported in Table 1, in our MASLD cohort 70% of patients were males, with a mean age of 53 ± 12 years. Prevalence of metabolic comorbidities was relevant: 86% of patients were overweight (mean BMI 28.8 ± 4.4 kg/m^2^), 91% dyslipidemic (mean LDL 116 ± 38 mg/dL; median triglycerides 135 [ 97–179] mg/dL), 56% hypertensive and 27% diabetic (mean glucose 104 ± 23 mg/dL). As for liver disease, median AST, ALT and GGT were 32 [24–41] U/L, 46 [31–68] U/L and 45 [26–82] U/L, respectively. At Fibroscan, 60% of the cohort presented with severe steatosis by CAP (mean CAP 290 ± 58 dB/m) and 17% had advanced hepatic fibrosis (i.e., ≥ F3) by LSM ≥ 8 kPa (median LSM 5.6 kPa [4.5–7.4] kPa). Finally, 14 patients (10%) presented ACLD, of whom 9 with a histological diagnosis and 5 non-invasively assessed. In the subset of 94 patients with paired liver biopsies, 38% presented with severe steatosis, 28% had MASH and 16% advanced fibrosis (≥ F3) (Supplementary Table S1). As for FIB4, median value was 1.17 [0.85–1.71].

**Table 1:** Baseline features according to baseline LAL/ptls ratio < or > median value in the whole cohort (*n* = 144)

	Total *N* = 144	LAL/ptls < median *N* = 72	LAL/ptls > median *N* = 72	*P* value
Sex, M, n (%)	101 (70)	47 (66)	54 (75)	0.275
Age, years	53 ± 12	54 ± 12	52 ± 12	0.336
LAL activity, nmol/spot/h	0.75 (0.50–0.97)	0.51 (0.40–0.64)	0.89 (0.75–1.16)	< 0.001
LAL/ptls ratio	0.33 (0.26–0.45)	0.26 (0.20–0.29)	0.45 (0.37–0.52)	< 0.001
BMI, kg/m^2^	28.8 ± 4.4	28.4 ± 4.8	29.2 ± 4.6	0.204
BMI ≥ 25 kg/m^2^, n (%)	124 (86)	60 (83)	64 (89)	0.471
Diabetes, n (%)	43 (30)	23 (32)	20 (27)	0.716
Glucose, mg/dl	104 ± 23	106 ± 32	103 ± 30	0.477
HOMA-IR index	3.7 (0.3–15.1)	4.0 (2.4–6.6)	3.7 (2.2–5.8)	0.278
Platelets, × 10^9^	216 (173–255)	210 (168–243)	220 (177–260)	0.249
Dyslipidemia, n (%)	131 (91)	66 (91)	65 (90)	1.000
Triglycerides, mg/dl	135 (97–179)	135 (102–183)	138 (94–178)	0.677
HDL-cholesterol, mg/dl	49 ± 13	48 ± 13	49 ± 13	0.665
LDL-cholesterol, mg/dl	116 ± 38	116 ± 35	117 ± 42	0.936
Hypertension, n (%)	81 (56)	40 (54)	41 (56)	0.867
AST, U/l	32 (24–41)	29 (24–39)	33 (23–42)	0.530
ALT, U/l	46 (31–68)	40 (30–68)	51 (33–72)	0.191
GGT, U/l	45 (26–82)	45 (24–73)	45 (26–99)	0.529
LSM, kPa	5.6 (4.5–7.4)	5.7 (4.5–7.5)	5.5 (4.5–7.3)	0.897
LSM ≥ 8 kPa, n (%)	24 (17)	14 (21)	10 (14)	0.500
CAP, dB/m	290 ± 58	290 ± 55	289 ± 62	0.940
CAP ≥ 280 dB/m, n (%)	86 (60)	47 (64)	39 (53)	0.259
MASH^a^, n (%)	26 (28)	10 (22)	16 (32)	0.113
NAS ≥ 5^a^, n (%)	37 (39)	18 (40)	19 (39)	0.674
Fibrosis ≥ F2^a^, n (%)	39 (41)	19 (42)	20 (41)	0.676
ACLD, n (%)	14 (10)	10 (14)	4 (6)	0.158

ACLD: advanced chronic liver disease; ALT, alanine aminotransferase; AST, aspartate aminotransferase; BMI, body mass index; CAP, controlled attenuation parameter; GGT, gamma-glutamyl transferase; HOMA-IR, homeostatic model assessment insulin resistance; LAL, lysosomal acid lipase; LAL/ptls, LAL/platelets ratio; LSM, liver stiffness measurement; MASH, metabolic-dysfunction associated steatohepatitis; NAS, NAFLD activity score

Data are expressed as mean ± standard deviation or median (interquartile range). Categorical variables are expressed as number and percentage

^a^Only available in 94 patients with paired biopsies (LAL < median = 45; > median = 49)

### Baseline features according to LAL/platelet ratio

At baseline, patients were stratified according to the median value of the LAL/ptls ratio (0.33, [0.26–0.45] nmol/spot/h). The two groups were superimposable in terms of metabolic and liver parameters. In particular, no differences in histological features were observed between patients with a LAL/ptls ratio above or below the median (Table 1).

### Changes in metabolic features over time

As reported in Supplementary Table 2, mean BMI significantly decreased at follow-up compared to baseline (28.8 ± 4.4 vs. 27.9 ± 4.5 kg/m^2^, *p* < 0.001); accordingly, the proportion of overweight individuals declined from 86 to 70% (*p* < 0.001). Conversely, prevalence of diabetes and hypertension increased from 30 to 38% (*p* = 0.012) and from 56 to 64% (*p* = 0.023), respectively, with a parallel increase in the use of anti-diabetic and anti-hypertensive medications.

Prevalence of dyslipidemia showed a trend toward a reduction in prevalence (91% vs. 89%, *p* = 0.060), with significantly lower plasma levels of triglycerides and LDL-cholesterol (135 [97.5–179] vs. 115 [82.5–153.5] mg/dL, *p* < 0.001; 116 ± 38 vs. 87 ± 37 mg/dL, *p* < 0.001) from baseline to follow-up, possibly consequent to an increased use of lipid-lowering drugs (statins: 23% vs. 49%, *p* < 0.001; ezetimibe: 4% vs. 21%, *p* < 0.001).

When analyzing the subset of patients with paired liver biopsy, results remained consistent (Supplementary Table S3).

### Changes in hepatic features over time

Baseline histological features of the 94 patients with paired liver biopsies are reported in Supplementary Table 1. In this subset, 10% of patients experienced a worsening of NAS of at least 1 point, 10% developed MASH and 10% showed worsening of at least 1 fibrosis stage. Only 7 patients worsened NAS score ≥ 2 points and 4 developed advanced fibrosis stages over time (≥ F3).

Overall, 26% of patients met the composite histological endpoint (i.e., development of MASH and/or worsening of NAS ≥ 1 point and/or fibrosis ≥ 1 stage) (Table 2).

**Table 2:** Changes of histological hepatic features at follow-up in the subset of patients with paired biopsies (*n* = 94)

Histological features	Change over time
MASH	Development *n* = 9 (10%) Stable *n* = 84 (89%) Resolution *n* = 1 (1%)
NAS (at least 1 point)	Worsening *n* = 9 (10%) Stable *n* = 83 (88%) Improvement *n* = 2 (2%)
Fibrosis (at least 1 stage)	Worsening *n* = 9 (10%) Stable *n* = 78 (83%) Improvement *n* = 7 (7%)
Composite histological endpoint (development of MASH and/or worsening of NAS and/or worsening of at least 1 stage fibrosis)	Yes *n* = 24 (26%) No *n* = 70 (74%)

MASH, metabolic-dysfunction associated steatohepatitis; NAS, NAFLD activity score

Conversely, in the whole cohort, LSM and CAP values remained overall stable over time (median LSM 5.6 [4.5–7.4] vs. 5.6 [4.8–7.7] kPa, *p* = 0.154; CAP 290 ± 58 vs. 282 ± 49 dB/m, *p* = 0.255). Similarly, no difference in transaminases was observed between baseline and follow-up (Supplementary Table 2). On the contrary, FIB-4 values significantly progressed raising from 1.17 [0.85–1.71] to 1.37 [1.02–2.12] (*p* < 0.001). Finally, the prevalence of cirrhosis slightly but significantly increased (10% vs. 13%, *p* = 0.025).

### Association between baseline LAL/ptls ratio and disease progression

**Fig. 1:**
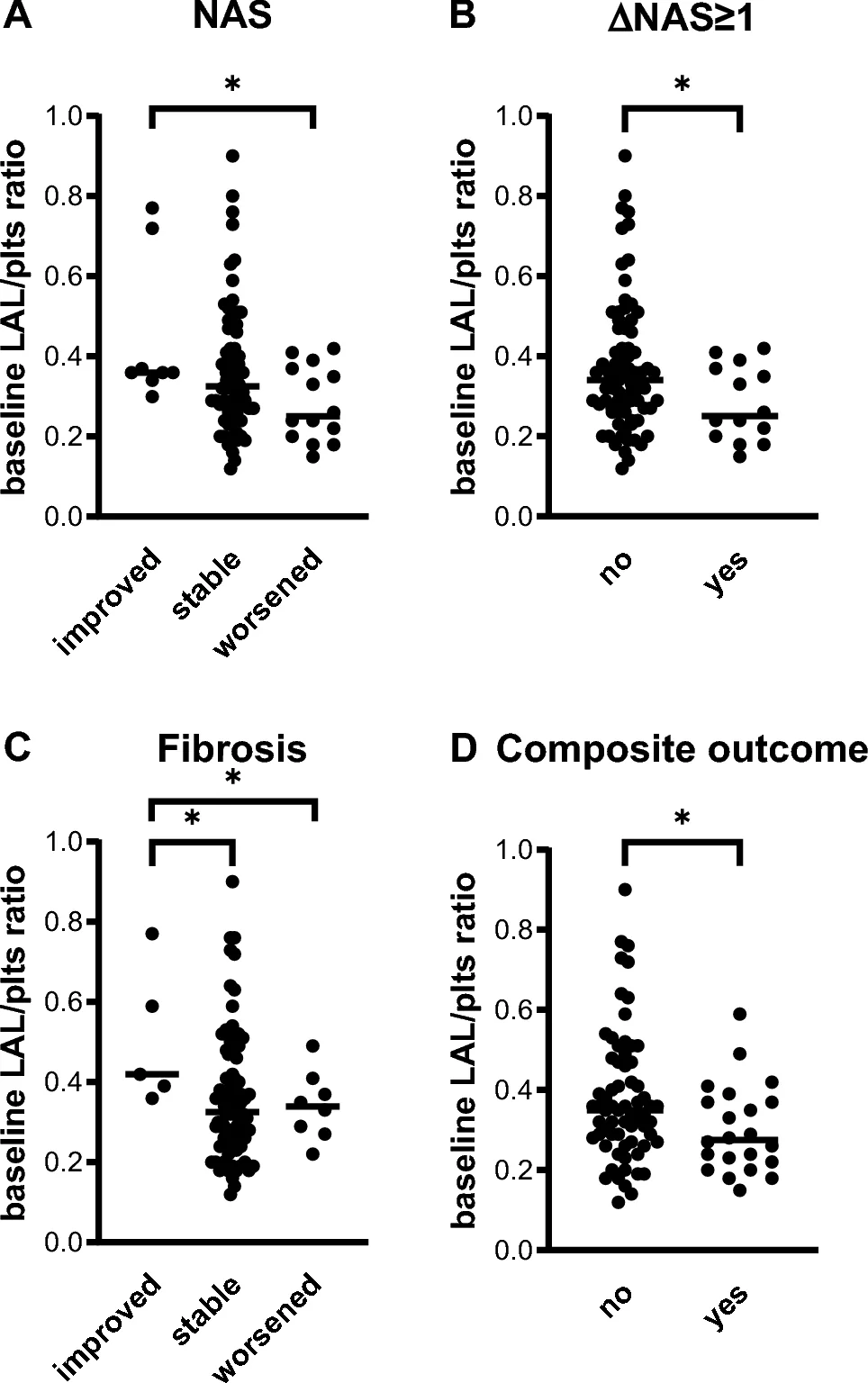
LAL/platelet ratio at baseline and changes in histopathological features at follow-up. Panel A. One or more points decrease (improved) or increase (worsened) in NAFLD activity score (NAS) from baseline to follow-up in patients with paired biopsies (*n* = 94). Panel B. Patients with (yes) or without (no) increase of NAS ≥ 1 point at follow-up. Panel C. One or more stages decrease (improved) or increase (worsened) in fibrosis from baseline to follow-up in patients with paired biopsies (*n* = 94). Panel D. Patients with (yes) or without (no) the composite hepatic outcome, defined as development of metabolic-dysfunction associated steatohepatitis (MASH) and/or worsening of fibrosis ≥ 1 stage and/or worsening of NAS ≥ 1 points. *P < 0.05

In patients with paired biopsies, a lower baseline LAL/ptls ratio was significantly associated with histological deterioration (Fig. 1). Specifically, we observed significantly lower baseline LAL/ptls values in patients with worsening of at least 1 point in NAS score (0.25 [0.19–0.37] vs. 0.34 [0.27–0.47] nmol/spot/h, *p* = 0.041), worsening of at least 1 fibrosis stage (0.33 [0.27–0.40] vs. 0.34 [0.26–0.46] nmol/spot/h, *p* = 0.045), or reaching the composite histological endpoint (0.27 [0.21–0.37] vs. 0.35 [0.24–0.47] nmol/spot/h, *p* = 0.045) (Table 4). Conversely, in the whole cohort, baseline LAL/ptls ratio was not associated with progression of liver disease assessed non-invasively by Fibroscan (i.e., worsening of CAP or LSM) (Table 3).

**Table 3:** Baseline LAL/ptls ratio and worsening of liver disease over time

	Baseline LAL/plts ratio	*P* value
Worsening of LSM (any kPa)		
No, *n* = 66	0.32 (0.26–0.46)	0.637
Yes, *n* = 78	0.34 (0.24–0.43)	
Worsening of LSM > 20%		
No, *n* = 107	0.32 (0.26–0.43)	0.309
Yes, *n* = 37	0.36 (0.26–0.52)	
Worsening of CAP (any dB/m)		
No, *n* = 86	0.31 (0.25–0.42)	0.693
Yes, *n* = 58	0.35 (0.22–0.47)	
Worsening of CAP > 20%		
No, *n* = 128	0.32 (0.24–0.43)	0.174
Yes, *n* = 16	0.41 (0.29–0.49)	
Development of MASH		
No, *n* = 85	0.33 (0.24–0.43)	0.175^a^
Yes, *n* = 9	0.37 (0.30–0.50)	
Worsening of NAS (any point)		
No, *n* = 85	0.34 (0.27–0.47)	0.041^a^
Yes, *n* = 9	0.25 (0.19–0.37)	
Worsening of fibrosis (at least 1 stage)		
No, *n* = 85	0.34 (0.26–0.46)	0.045^a^
Yes, *n* = 9	0.33 (0.27–0.40)	
Composite histological endpoint^b^		
No, *n* = 70	0.35 (0.24–0.47)	0.045^a^
Yes, *n* = 24	0.27 (0.21–0.37)	

CAP, controlled attenuation parameter; LSM, liver stiffness measurement; MASH, metabolic-dysfunction associated steatohepatitis; NAS, NAFLD activity score

Data are expressed as median (IQR)

^a^Only in patients with paired biopsies (n = 94)

^b^Composite histological endpoint defined as development of MASH and/or worsening of NAS and/or worsening of at least 1 stage fibrosis

### Predictors of histological progression

As depicted in Table 4, patients with the composite histological endpoint had significantly lower baseline LAL/ptls ratio (0.27 [0.21–0.37] vs. 0.35 [0.24–0.47] nmol/spot/h, *p* = 0.045), higher HOMA-IR (6.6 [3.7–8.5] vs. 3.9 [2.4–6.5], *p* = 0.050), higher baseline CAP (321 ± 45 vs. 285 ± 60 dB/m, *p* = 0.006), and higher LSM (6.8 [5.4–7.9] vs. 5.3 [4.4–6.8] kPa, *p* = 0.006) compared to those who did not met the composite histological endpoint at follow-up.

**Table 4:** Baseline features according to worsening or not worsening of histology as composite endpoint in the subset of patients with paired biopsies (*n* = 94)

Baseline features	Total *n* = 94	No composite endpoint *n* = 70	Yes composite endpoint *n* = 24	*P* value
Sex, M, n (%)	66 (71)	51 (73)	15 (62)	0.426
Age, years	52 ± 11	53 ± 11	50 ± 11	0.391
LAL/ptls ratio	0.34 (0.26–0.42)	0.35 (0.24–0.47)	0.27 (0.21–0.37)	0.045
BMI, kg/m^2^	29.2 ± 4.3	29.1 ± 4.2	29.6 ± 4.9	0.678
BMI ≥ 25 kg/m^2^, n (%)	84 (89)	61 (87)	23 (95)	0.471
Diabetes, n (%)	30 (31)	20 (28)	10 (42)	0.306
HOMA-IR index	4.4 (2.7–7.2)	3.9 (2.4–6.5)	6.6 (3.7–8.5)	0.050
Platelets, × 10^9^	224 (183–262)	224 (188–265)	225 (166–271)	0.831
Dyslipidemia, n (%)	88 (94)	64 (92)	24 (100)	0.324
Hypertension, n (%)	55 (58)	38 (55)	17 (73)	0.211
AST, U/l	33 (25–44)	30 (25–42)	36 (28–51)	0.313
ALT, U/l	52 (34–72)	50 (30–74)	55 (34–73)	0.958
GGT, U/l	44 (28–73)	44 (28–73)	47 (28–81)	0.588
LSM, kPa	5.6 (4.8–7.6)	5.3 (4.4–6.8)	6.8 (5.4–7.9)	0.006
LSM ≥ 8 kPa, n (%)	17 (18)	11 (15)	6 (26)	0.306
CAP, dB/m	293 ± 60	285 ± 60	321 ± 45	0.006
CAP ≥ 280 dB/m, n (%)	59 (63)	38 (55)	21 (86)	0.017
MASH, n (%)	26 (28)	19 (28)	7 (32)	0.791
NAS ≥ 5, n (%)	37 (39)	27 (30)	10 (44)	0.468
Fibrosis ≥ F2, n (%)	39 (41)	27 (38)	12 (50)	0.325

ALT, alanine aminotransferase; AST, aspartate aminotransferase; BMI, body mass index; CAP, controlled attenuation parameter; GGT, gamma-glutamyl transferase; HOMA-IR, homeostatic model assessment insulin resistance; LAL, lysosomal acid lipase; LAL/ptls, LAL/platelets ratio; LSM, liver stiffness measurement; MASH, metabolic-dysfunction associated steatohepatitis; NAS, NAFLD activity score

Data are expressed as mean ± standard deviation or median (interquartile range). Categorical variables are expressed as number and percentage

The composite histological endpoint was defined as development of MASH and/or worsening of NAS and/or worsening of at least 1 stage fibrosis

At multivariate logistic regression, LAL/ptls ratio (log transformed for the analysis) remained independently associated with the composite endpoint (OR 0.51, 95% CI 0.04–0.60, *p* = 0.018), together with HOMA-IR (OR 1.32, 95% CI 1.00–1.74, *p* = 0.050). This association persisted even when substituting CAP with LSM in the model (OR 0.045, 95% CI 0.003–0.693, *p* = 0.026) or when excluding patients with paired biopsy and ACLD at baseline (*n* = 6 patients) (Table 5).

**Table 5:** Multivariate analysis of baseline factors associated with the histological composite endpoint

	OR	CI 95%	*P* value
Age	0.92	0.84–1.00	0.079
Sex, female	1.15	0.16–8.26	0.884
LAL/ptls ratio, ln	0.51	0.04–0.60	0.018^*^
HOMA-IR index	1.32	1.00–1.74	0.050
CAP	3.90	0.60–25.2	0.153

CAP, controlled attenuation parameter; HOMA-IR, homeostatic model assessment insulin resistance; LAL/ptls, lysosomal acid lipase/platelets ratio; ln, natural logarithm; OR, odds ratio; CI, confidence interval

^*^when excluding ACLD patients from analysis: OR 0.69, 95% CI 0.06–0.80, *p* = 0.033

When LAL/ptls was analyzed by tertiles (lower and higher cutoff 0.28 and 0.38 nmol/spot/h), a significant overall association with histological progression was observed (*p* for overall effect = 0.027). In particular, patients in the lowest tertile showed a markedly higher risk compared with the highest tertile, although confidence intervals were wide due to the limited number of events (OR 17.5, CI 95% 1.85–167, *p* = 0.013).

**Fig. 2:**
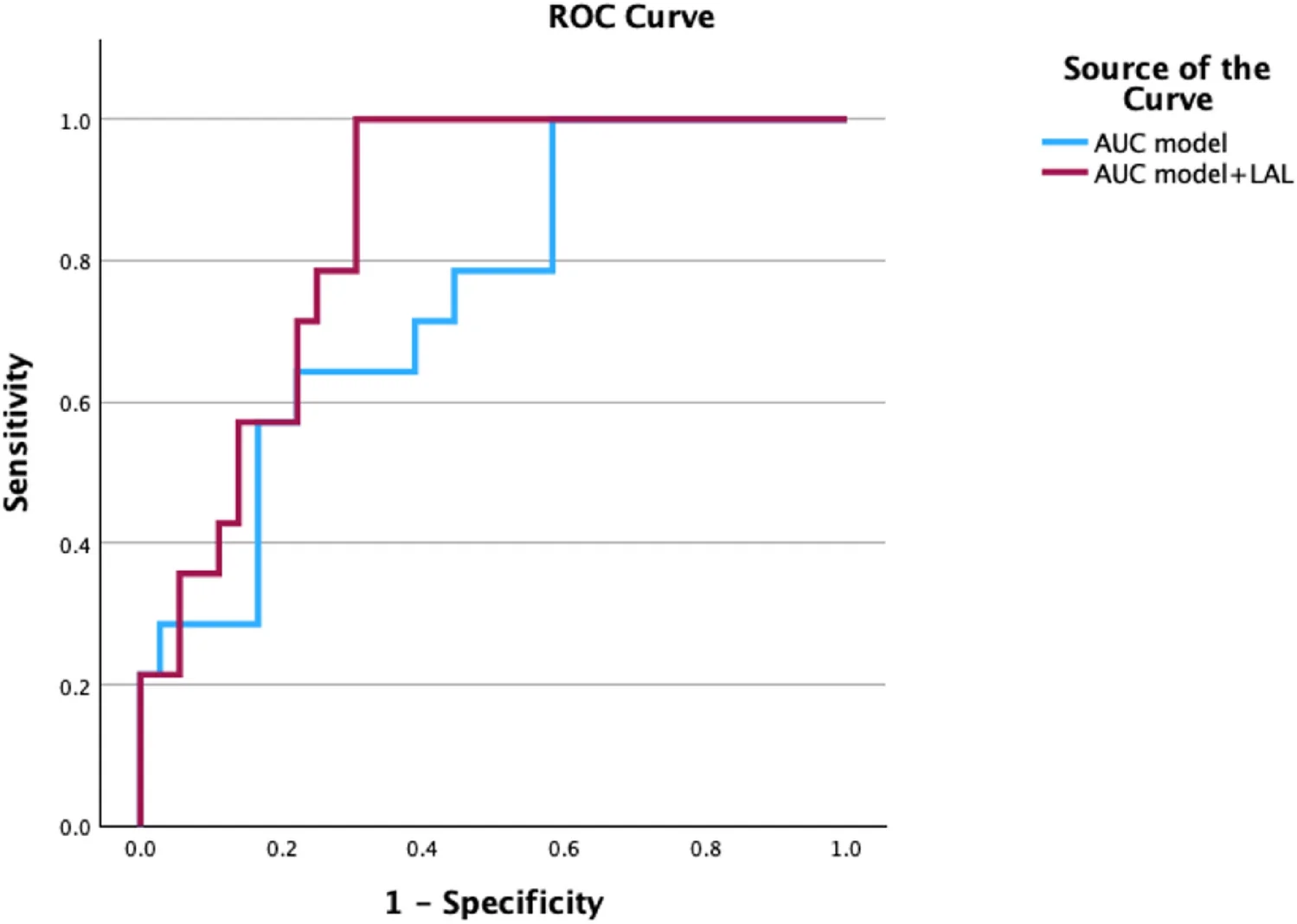
Receiver operating characteristic (ROC) curves of the clinical models including age, sex, HOMA and CAP without (blue line) or with (purple line) LAL/ptls for prediction of the histological composite endpoint

Finally, receiver operating characteristic (ROC) curve analysis was performed to evaluate the incremental predictive value of LAL/ptls. The addition of LAL/ptls to the clinical model including only variables significantly associated or with intrinsic physiological impact on the histological endpoint (i.e., age, sex, HOMA-IR and CAP) improved discrimination for the histological composite endpoint (AUC 0.849 vs. 0.750) (Fig. 2).

## Discussion

In this prospective single-center study with a median follow-up of more than 8 years, we demonstrate that lower baseline lysosomal acid lipase (LAL) activity normalized for platelet count identifies MASLD patients at increased risk of histological disease progression. In particular, reduced LAL/platelet ratio was associated with worsening in NAS, development of MASH, and progression of fibrosis after adjustment for established metabolic comorbidities. Interestingly, baseline LAL activity was not associated with progression of liver disease assessed non-invasively by Fibroscan, supporting the concept that LAL may capture biological processes, especially inflammation, that are not adequately reflected by current non-invasive tools. Furthermore, the availability of paired histological data for the majority of the cohort further strengthens the role of LAL activity as a predictor of progression. Notably, ROC analysis suggested that LAL/ptls may provide incremental prognostic information beyond routinely available clinical parameters, supporting its potential role in refining early risk stratification in MASLD.

Our results are consistent with accumulating evidence in the literature supporting an association between LAL activity and liver disease in MASLD. We previously demonstrated that patients with MASLD exhibit lower LAL activity compared with controls [10], a finding that was subsequently confirmed by a systematic review, including nine studies with more than 1,700 MASLD patients and 877 controls, which clearly reported an independent association between reduced LAL activity and MASLD [19]. Interestingly, other groups described the lowest LAL levels in cirrhotic patients [20–22]. Moreover, the association of LAL activity with histological features of MASLD, mainly inflammation, in our cohort is in line with a previous report showing that LAL levels were independently associated with increased necro-inflammatory activity, evaluated as NAS severity, in a series of 101 biopsy proven MASLD [23].

The clinical evidence we found relies on a strong biological background. Indeed, patients with genetic LAL deficiency present with rapidly progressive steatosis with marked inflammation and fibrosis, and LAL-deficient mice display extensive lipid accumulation, immune cell infiltration, and accelerated fibrogenesis by activation of hepatic stellate cells [24–26]. LAL is also critical for immune cell maturation and function, predisposing to a chronic pro-inflammatory state [27–29]. Despite not primarily highlighted by the current study, another possible mechanism of liver damage promoted by LAL is related to its critical role in efficient autophagy, which is needed for both hepatocyte and immune cell homeostasis, as impaired autophagic flux was shown to promote hepatocellular ballooning [26, 30]. Interestingly, we have previously demonstrated that PPAR agonists are able to rescue defective LAL activity caused by lipid accumulation, thus restoring autophagic flux [10, 31].

Alongside low LAL/ptls ratio, another predictor of damage progression was insulin-resistance, in line with what has already been reported in the literature [11]. However, patients with lower LAL/ptls ratios did not have higher HOMA-IR levels nor a higher prevalence of diabetes, possibly speculating on its role in the progression of liver disease beyond metabolic alterations.

In this series, we did not find any association between LAL/ptls and advanced liver disease at baseline, despite its predictive value for histological progression. This apparent contradiction likely reflects the different biological meanings of cross-sectional vs. longitudinal assessments. While advanced fibrosis represents a late structural stage, reduced LAL/ptls may capture earlier metabolic–inflammatory alterations that predispose to disease worsening, suggesting that LAL/ptls may act primarily as a dynamic marker of disease susceptibility rather than a marker of established advanced liver damage.

Some limitations deserve consideration. First, the study was single-center and the number of patients with paired biopsies, while relatively large compared to similar studies, remains limited, which may reduce the power to detect subtler associations. Moreover, the small number of patients meeting more stringent histological thresholds (e.g., NAS increase ≥ 2 or progression to advanced fibrosis ≥ F3) limited the feasibility of further sensitivity analyses. In addition, the limited number of histological events together with the log-transformation of LAL/ptls may have accounted for the width and asymmetry of the confidence intervals and warrant cautious interpretation of the findings. Second, biopsies were performed for clinical indications, potentially introducing selection bias toward subjects with more active disease. Third, LAL activity on DBS is influenced by platelet count, although this issue was mitigated using the LAL/platelet ratio, which better reflects the enzyme’s biological variability in liver disease. Even if LAL activity was measured on circulating cells, we have previously shown a correlation with enzymatic activity measured on liver biopsies in this cohort [10]. However, this correlation indicates that acquired LAL deficiency in MASLD occurs not only in the liver but also in circulating immune cells, possibly contributing to liver disease progression. In addition, our results were confirmed even after exclusion of patients with ACLD. Third, despite the independent association, the observational nature of the study prevents causal inference. Finally, unmeasured changes in lifestyle and pharmacological treatments during follow-up may have introduced residual confounding, as these time-varying factors could affect both LAL/ptls and liver histology. Although baseline covariates were accounted for, this possibility cannot be fully excluded.

However, it is worth pointing out that the relationship between LAL activity and liver disease progression is supported by biological plausibility, as discussed above.

Despite these limitations, the strength of this study is the availability of prospective histological data in a substantial proportion of patients, conversely to available data in literature on this topic which come from cross-sectional studies.

In conclusion, low LAL activity could identify MASLD patients at increased risk of histological progression, particularly in terms of inflammatory activity and fibrosis. LAL activity may represent a useful biological marker that complements non-invasive tools by capturing processes that are not reflected by VCTE-derived parameters, as inflammation, helping to identify patients requiring closer surveillance or earlier and stricter intervention. In particular, from a translational standpoint, our findings suggest that LAL/ptls may represent a complementary biomarker to refine risk stratification in MASLD. Although clinically actionable thresholds cannot yet be defined, the observed improvement in ROC discrimination after addition of LAL/ptls to the base clinical model supports its potential incremental value alongside existing non-invasive tools. In our series, we identified a cut-off of 0.28 nmol/spot/h that might be proposed as a threshold to be validated in future prospective studies. Therefore, incorporation of LAL/ptls into current algorithms may improves patient-level risk prediction as well as exploring whether interventions capable of restoring LAL activity, as the use of PPAR agonists, may offer therapeutic benefit in patients with MASLD.

## Supplementary Information

Below is the link to the electronic supplementary material.

Supplementary file1 (DOCX 27 KB)

## Data Availability

The datasets generated and/or analyzed during the current study are not publicly available due to privacy and ethical restrictions but are available from the corresponding author on reasonable request.
