# Supplementary material for: Low lysosomal acid lipase activity is associated with histological progression of metabolic dysfunction-associated steatotic liver disease
Source: Hepatol Int. 2026 Apr 22;20(4):1073–83. doi: 10.1007/s12072-026-11084-6 (PMC13518429; doi:10.1007/s12072-026-11084-6)
Supplement: Supplementary file 1 [file 12072_2026_11084_MOESM1_ESM.docx]

Supplementary table 1. Baseline histological features of the subset of patients with paired biopsies (n=94)

| Histological features | n (%) |
| --- | --- |
| Steatosis  Grade I, n (%)  Grade II, n (%)  Grade III, n (%) | 27 (29)  31 (33)  36 (38) |
| NAS  1, n (%)  2, n (%)  3, n (%)  4, n (%)  5, n (%)  6, n (%)  7, n (%) | 8 (9)  11 (12)  30 (32)  8 (9)  29 (25)  9 (9)  4 (4) |
| NAS ≥5, n (%) | 37 (39) |
| Ballooning grade  0, n (%)  1, n (%)  2, n (%) | 59 (63)  33 (35)  2 (2) |
| MASH, n (%) | 26 (28) |
| Fibrosis stage  0, n (%)  1, n (%)  2, n (%)  3, n (%)  4, n (%) | 21 (22)  34 (36)  24 (25)  14 (15)  1 (1) |
| Fibrosis ≥F2, n (%) | 39 (41) |

Supplementary table 2. Changes of metabolic and hepatic features between baseline and follow-up in the whole cohort (n=144)

|  | Baseline  n=144 | Follow-up  n=144 | *P* value |
| --- | --- | --- | --- |
| BMI, kg/m^2^ | 28.8±4.4 | 27.9±4.5 | **<0.001** |
| BMI ≥25 kg/m^2^, n (%) | 124 (86) | 101 (70) | **<0.001** |
| Diabetes, n (%) | 43 (30) | 55 (38) | **0.012** |
| Glucose, mg/dl | 104±23 | 108±23 | **0.037** |
| HOMA-IR index | 3.7 (0.3-15.1) | 5.2 (2.2-13.3) | 0.105 |
| Anti-diabetic drugs, n (%)  Metformin, n (%)  Insulin, n (%)  GLP-1RA, n (%)  SGLT2, n (%) | 28 (19)  25 (17)  7 (5)  0 (0)  0 (0) | 48 (33)  36 (25)  8 (6)  17 (12)  7 (5) | **<0.001**  **0.003**  0.450  **<0.001**  **<0.001** |
| Platelets, x10^9^ | 216 (173-255) | 216 (159-261) | 0.870 |
| Triglycerides, mg/dl | 135 (97-179) | 115 (82-153) | **<0.001** |
| HDL-cholesterol, mg/dl | 49±13 | 49±12 | 0.107 |
| LDL-cholesterol, mg/dl | 116±38 | 87±37 | **<0.001** |
| Dyslipidemia, n (%) | 131 (91) | 128 (89) | 0.060 |
| Statins, n (%) | 33 (23) | 71 (49) | **<0.001** |
| Hypertension, n (%) | 81 (56) | 92 (64) | **0.023** |
| Anti-hypertensive drugs, n (%) | 76 (53) | 88 (61) | **0.005** |
| AST, U/l | 32 (24-41) | 29 (23-36) | 0.164 |
| ALT, U/l | 46 (31-68) | 38 (24-48) | 0.165 |
| GGT, U/l | 45 (26-82) | 38 (22-88) | 0.052 |
| FIB-4 | 1.17 (0.85-1.71) | 1.37 (1.02-2.12) | **<0.001** |
| LSM, kPa | 5.6 (4.5-7.4) | 5.6 (4.8-7.7) | 0.154 |
| LSM ≥8 kPa, n (%) | 24 (17) | 35 (24) | 0.090 |
| CAP, dB/m | 290±58 | 282±49 | 0.255 |
| CAP ≥280 dB/m, n (%) | 86 (60) | 76 (53) | 0.286 |
| ACLD*, n (%) | 14 (10) | 19 (13) | 0.025 |

Data are expressed as mean ± standard deviation or median (interquartile range). Categorical variables are expressed as number and percentage. *of whom 9 histologic and 5 clinical diagnoses at baseline, 10 histologic and 9 clinical diagnoses at follow-up.

ABBREVIATIONS: ACLD: advanced chronic liver disease; ALT, alanine aminotransferase; AST, aspartate aminotransferase; BMI, body mass index; CAP, controlled attenuation parameter; GGT, gamma-glutamyl transferase; HOMA-IR, homeostatic model assessment insulin resistance; LSM, liver stiffness measurement.

Supplementary table 3. Changes of metabolic and hepatic features between baseline and follow-up in the cohort of patients with paired biopsy (n=94)

|  | Baseline  n=94 | Follow-up  n=94 | *P* value |
| --- | --- | --- | --- |
| BMI, kg/m^2^ | 29.3±4.3 | 28.4±4.2 | **0.002** |
| BMI ≥25 kg/m^2^, n (%) | 84 (89) | 72 (77) | **0.003** |
| Diabetes, n (%) | 33 (35) | 44 (47) | **0.022** |
| Glucose, mg/dl | 105±23 | 111±25 | **0.025** |
| HOMA-IR index | 4.4 (2.7-7.2) | 5.4 (3.2-7.6) | 0.163 |
| Anti-diabetic drugs, n (%) | 20 (21) | 36 (38) | **<0.001** |
| Platelets, x10^9^ | 224 (183-262) | 218 (167-269) | **<0.001** |
| Triglycerides, mg/dl | 141 (104-187) | 129 (87-166) | **0.005** |
| HDL-cholesterol, mg/dl | 48±14 | 54±29 | 0.061 |
| LDL-cholesterol, mg/dl | 121±41 | 84±39 | **<0.001** |
| Dyslipidemia, n (%) | 90 (96) | 86 (92) | 0.083 |
| Statins, n (%) | 24 (26) | 50 (53) | **<0.001** |
| Hypertension, n (%) | 54 (58) | 63 (67) | **0.033** |
| Anti-hypertensive drugs, n (%) | 53 (56) | 62 (66) | **0.007** |
| AST, U/l | 33 (25-44) | 29 (23-37) | 0.132 |
| ALT, U/l | 52 (33-72) | 36 (25-51) | **<0.001** |
| GGT, U/l | 44 (28-73) | 38 (25-70) | 0.132 |
| FIB-4 | 1.17 (0.87-1.60) | 1.37 (1.00-1.86) | **0.010** |
| LSM, kPa | 5.6 (4.5-7.6) | 6.3 (4.9-8.3) | 0.225 |
| LSM ≥8 kPa, n (%) | 18 (19) | 24 (25) | 0.225 |
| CAP, dB/m | 293±59 | 287±46 | 0.419 |
| CAP ≥280 dB/m, n (%) | 60 (64) | 51(54) | 0.223 |
| MASH, n (%) | 26 (28) | 34 (36) | **0.035** |
| NAS ≥5, n (%) | 37 (39) | 39 (41) | 0.564 |
| Fibrosis ≥F2, n (%) | 39 (41) | 46 (49) | 0.180 |
| ACLD, n (%) | 6 (6) | 8 (8) | 0.852 |
